# Predicting Metabolite–Disease Associations Based on LightGBM Model

**DOI:** 10.3389/fgene.2021.660275

**Published:** 2021-04-13

**Authors:** Cheng Zhang, Xiujuan Lei, Lian Liu

**Affiliations:** School of Computer Science, Shaanxi Normal University, Xi’an, China

**Keywords:** metabolite-disease associations, light gradient boosting machine, features, computational method, performance evaluation

## Abstract

Metabolites have been shown to be closely related to the occurrence and development of many complex human diseases by a large number of biological experiments; investigating their correlation mechanisms is thus an important topic, which attracts many researchers. In this work, we propose a computational method named LGBMMDA, which is based on the Light Gradient Boosting Machine (LightGBM) to predict potential metabolite–disease associations. This method extracts the features from statistical measures, graph theoretical measures, and matrix factorization results, utilizing the principal component analysis (PCA) process to remove noise or redundancy. We evaluated our method compared with other used methods and demonstrated the better areas under the curve (AUCs) of LGBMMDA. Additionally, three case studies deeply confirmed that LGBMMDA has obvious superiority in predicting metabolite–disease pairs and represents a powerful bioinformatics tool.

## Introduction

Metabolism is a series of ordered chemical reactions, which has a significant influence on biological life maintenance, such as the organism’s growth, reproduction, and reaction to the external environment (Dunn and Ellis, 2005). Metabolic processes are usually divided into two types. The first type is decomposing large molecules to acquire energy, such as cell respiration, while the other type is utilizing energy for the synthesis of each part inside the cells, such as nucleic acids and proteins (Cheng et al., 2017). In unhealthy conditions, relevant products in metabolism (metabolites) will be abnormal, which indicates that finding more disease-related metabolites is beneficial to disease prevention and treatment (Boja et al., 2014). Consequently, it is of high importance to identify the relationship among metabolites and diseases.

Although some traditional techniques of metabolomics has prompted their analysis and development, such as nuclear magnetic resonance (NMR) spectroscopy or liquid/gas chromatography-mass spectrometry (LC/GC-MS) (Xianlin et al., 2011; Tang et al., 2014), the proportion of undiscovered associations between metabolites and diseases is still high. Moreover, some limitations exist, such as the time and funds required to mine disease-related metabolites in biological experiments. Therefore, effective computational methods for predicting disease-related metabolites are attracting more and more attention, which is beneficial to promoting the development to discover potential metabolite–disease associations. The idea of Random Walk with Restart for MiRNA-Disease Association (RWRMDA) (Hu et al., 2018) is to construct a metabolite–metabolite functional similarity network and implement RWR from known disease-related metabolite seed nodes to prioritize potential disease-related ones. However, this method uses less information for diseases or metabolites when calculating similarities, and its predictive performance needs to be improved.

**FIGURE 1:**
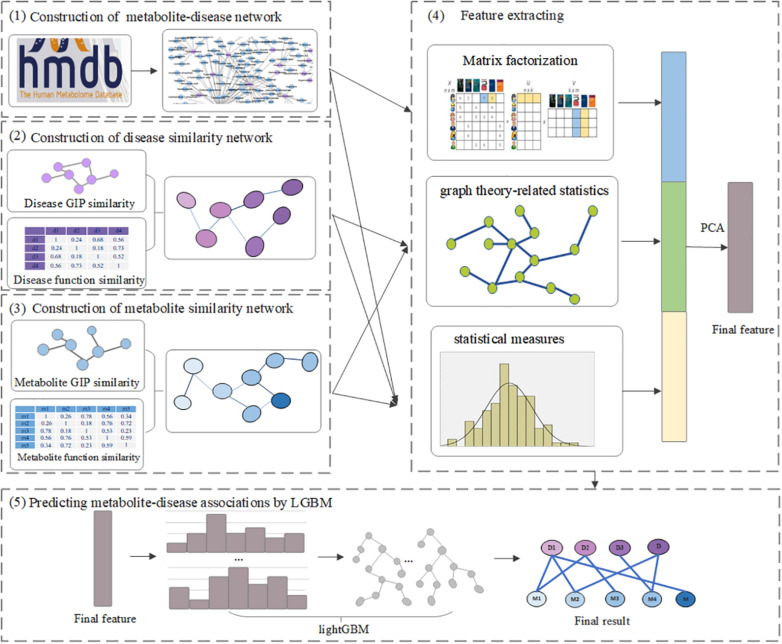
The flowchart of LGBMMDA.

In this article, we present a computational method, LGBMMDA, based on Light Gradient Boosting Machine (LightGBM) (Ke et al., 2017), to identify metabolite–disease associations (Figure 1). Firstly, we extract the data of metabolite-related pathways as part of the integrated similarity network. Secondly, features are selected from the relevant similarity network and known metabolite–disease associations using the statistical measures, graph theoretical measures, and matrix factorization measures. Furthermore, the principal component analysis (PCA) (Deutsch, 2004) algorithm, which is a technique for analyzing and simplifying datasets, is utilized to extract deep features. Thirdly, the LightGBM classifier is utilized to obtain the potential association scores. In addition, the LOOCV and fivefold cross-validation are adopted to evaluate the performance of LGBMMDA, which achieves 0.9738 and 0.9715, respectively. Besides, three types of case studies for common diseases are carried out to evaluate the ability of the method to predict metabolites. These aforementioned experiments show that LGBMMDA is a reliable and excellent model to infer unknown metabolites–diseases associations.

## Materials and Methods

### Human Metabolite–Disease Associations

**FIGURE 2:**
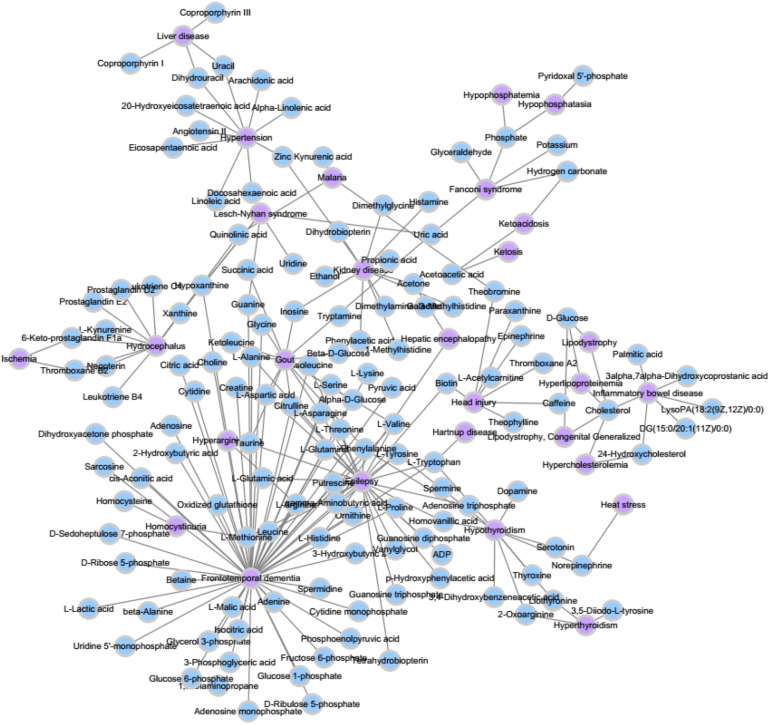
A part of known metabolite–disease association network.

We extracted the experimentally confirmed human metabolite–disease associations from the last updated database (HMDB) (Wishart et al., 2017). Then, we performed the following steps on these associations: Firstly, the disease-related symptoms from the human symptom–disease network (HSDN) (Zhou et al., 2014; Ma et al., 2016) are used to calculate disease similarity after repeated associations; thus, the diseases that do not exist in the HSDN are removed. Secondly, the metabolite-related pathways from HMDB become part of the metabolite similarities, such that we keep the metabolites that are relevant to the diseases we selected. Finally, we obtain 127 diseases and 794 metabolites, which have 1,908 experimentally human metabolite–disease associations (see Figure 2). The parameters *nm* and *nd* represent the number of metabolites and diseases, respectively. Matrix *M* represents the adjacency matrix of metabolite–disease associations, such that the entity *M*(*i*,*j*) in row *i* and column *j* is 1 if disease *i* is associated with metabolite *j* and 0 otherwise.

### Metabolite Functional Similarity

According to the hypothesis that metabolites with similar functions have a higher probability of possessing similar pathways, we utilize the Hamming similarity (Charikar, 2002) to measure the functional similarity of two metabolites by considering their related pathways. The metabolite functional similarity matrix is defined as *MHS*_(_*_*nm*_*_×_ *_*nm*_*_)_, such that the element value is calculated as follows (Zhang et al., 2020)

(1) $$M\,H\,S\,\left(m_{i},m_{j}\right)=1-\frac{\sum_{k=1}^{n\,p}M\,p\,V\,\left(M\,P\,\left(k,i\right),M\,P\,\left(k,j\right)\right)}{n\,s}$$

(2) $$MpV(MP\left(k,i\right),MP\left(k,j\right))=\;\{\begin{array}{c} \;1,\;if\;the\;values\;of\;MP\left(k,i\right)\;and\;MP\left(k,j\right)\;are\;different\;\\ 0,\;ifthe\;values\;of\;MP\left(k,i\right)\;and\;MP\left(k,j\right)\;are\;same \end{array}$$

where *MHS*(*m*_*i*_,*m*_*j*_) represents the Hamming similarity between metabolite node *m_i* and *m_j*; *np* denotes the number of pathways. If there are existing associations between the metabolite *i* and pathway *k*, *MP*(*k*,*i*) is set to 1 in metabolite-pathway association network (*MP*).

### Disease Functional Similarity

Considering the assumption that two diseases with similar functions usually have similar symptoms, the values of two disease-related symptom sets are used to obtain their functional similarities. Let the sets Sda={Sda⁢(1),Sda⁢(2),Sda⁢(a⁢s)} and sets Sdb={Sdb⁢(1),Sdb⁢(2),Sdb⁢(b⁢s)}describe the symptom sets of diseases *a* and *b*, where *as* and *bs* denote the number of symptoms associated with diseases *a* and *b*, respectively. Firstly, we calculate the information entropy of Sda as follows (Gu et al., 2017)

(3) H⁢(Sda)=-∑i=1n⁢sp⁢(Sda⁢(i))⁢{log2⁡p⁢(Sda⁢(i))}

(4) p⁢(Sda⁢(i))=n⁢(Sda⁢(i))T⁢n

where *Tn* denotes the number of disease-symptom associations, n⁢(Sda⁢(i)) is the number of the *i*th symptom related with disease *a* in the disease-symptom set,p⁢(Sda⁢(i))represents the frequency about the *i*th symptom associated with disease *a*, and H⁢(Sda) is the information entropy of Sda. The normalized mutual information (NMI) of Sda and Sdb is used to measure the functional similarity between disease *a* and *b* as follows:

(5) D⁢N⁢F⁢(da,db)=2⁢H⁢(Sda⁢⋂Sdb)H⁢(Sda)+H⁢(Sdb)

where matrix *DNF* represents the functional similarity matrix; Sda, Sdb, and H⁢(Sda⁢⋂Sdb) denote the information entropy of Sda, Sdb and the intersection set of Sda and Sdb, respectively.

### Gaussian Interaction Profile Kernel Similarity

Following literature (Gu et al., 2017) the GIP kernel for the similarities about diseases and metabolites captures the key features of the metabolite–disease association data. Calculating such kind of similarities is based on the assumption that similar diseases are more likely to contain functionally similar metabolites, and vice versa. Let the binary vector *V*(*d*_*i*_), which is the row vector of the matrix *M* where the disease *d_i* is located, represent the interaction profiles of disease *d_i*. Then, the relevant similarities for diseases *DGS*(*d_i*,*d_j_*) between the diseases *d_i* and *d_j* can be shown as follows:

(6) $$D\,G\,S\,\left(d_{i},d_{j}\right)=e\,x\,p\,\left(-\omega_{d}\,{||V\,\left(d_{i}\right)-V\,\left(d_{i}\right)||}^{2}\right)$$

(7) $$\omega_{d}=\omega_{d}^{\prime }/\left(\frac{1}{n\,d}\,\sum_{i=1}^{n\,d}{||V\,\left(d_{i}\right)||}^{2}\right)$$

where ω_*d*_ is a parameter that controls the kernel bandwidth, acquired by normalizing the new bandwidth parameter ω′d. Similarly, the GIP kernel of the similarities*MGS*(*m*_*i*_,*m*_*j*_) between metabolites *m_i* and *m_j* is defined as follows:

(8) $$M\,G\,S\,\left(m_{i},m_{j}\right)=e\,x\,p\,\left(-\omega_{d}\,{||V\,\left(m_{i}\right)-V\,\left(m_{j}\right)||}^{2}\right)$$

(9) $$\omega_{m}=\omega_{m}^{\prime }/\left(\frac{1}{n\,m}\,\sum_{i=1}^{n\,m}{||V\,\left(m_{i}\right)||}^{2}\right)$$

where ω_*m*_ is a parameter that controls the kernel bandwidth, acquired by normalizing the new bandwidth parameter ωm′.

### Integrated Similarity for Metabolites and Diseases

In order to ensure that similarity information exists for every pair in metabolites or diseases, we integrated the disease functional similarities with GIP kernel similarities, which is shown as follows:

(10) $$I\,D\,S\,\left(d_{i},d_{j}\right)=\left\{ \begin{array}{c} D\,N\,S\,\left(d_{i},d_{j}\right)\;i\,f\,D\,N\,S\,\left(d_{i},d_{j}\right)\neq 0\\ D\,G\,S\,\left(d_{i},d_{j}\right)\;o\,t\,h\,e\,r\,w\,i\,s\,e \end{array}\right.$$

where *IDS*(*d*_*i*_,*d*_*j*_) represents the integrated disease similarities. Similarly, the integrated metabolite similarity matrix (*IMS*) is given as follows:

(11) $$I\,M\,S\,\left(m_{i},m_{j}\right)=\left\{ \begin{array}{c} F\,H\,S\,\left(m_{i},m_{j}\right)\,i\,f\,F\,H\,S\,\left(m_{i},m_{j}\right)\neq 0\\ M\,G\,S\,\left(m_{i},m_{j}\right)\,o\,t\,h\,e\,r\,w\,i\,s\,e \end{array}\right.$$

### Feature Extraction

Firstly, type 1 features (*F1*), which consist of the values of the sum, mean, and histogram distributions of metabolite/disease similarities, are calculated using the statistical measures for each disease/metabolite. We start by calculating the number of known associations in the relevant *i*th row/*j*th column of *M*. Then, the average of all similarity scores is computed according to the *i*th/*j*th row of *IDS*/*IMS.* Simultaneously, the similarity scores that ranges at [0, 1] are split into *n* parts (*n* = 5 in this work), and the proportion of similarity scores for *d(j)/m(i)* that fell into each part are counted as the histogram feature.

Secondly, type 2 features (*F2*) are calculated, which include the information about graph theory-related statistics. Before obtaining this type of features, we construct the unweighted graph, in which two nodes have an edge if their similarity score is beyond the mean value of all entities in *IDS*/*IMS*. Then, we extract the relevant neighbors’ information, betweenness, closeness, eigenvector centrality, and PageRank (Franceschet, 2010) scores of the disease/metabolite similarity network in an unweighted graph.

Thirdly, type 3 features (*F3*) are calculated. These features consist of the information about metabolite–disease pairs based on matrix factorization of *M.* The nonnegative matrix factorization (NMF) (Lee and Seung, 1999; Akbar et al., 2020), which was proposed by Lee and Seung, 1999, can help to solve the matrix sparsity problem. Thus, the metabolite–disease association matrix *M* can be factorized into two low-rank feature matrices A ∈ R^nm*k^ and B ∈ R^k*nd^, where *k* denotes the dimension of the metabolite and disease features in the low-rank spaces (*k* = 20).

Finally, the feature sets *F*(*i*,*j*) = [*F*1,*F*2,*F*3] for disease *i* and metabolite *j* is obtained. Meanwhile, PCA is applied to extract the more useful features.

## Light Gradient Boosting Machine

Some boosting algorithms, such as the Gradient Boosting Decision Tree (GBDT) and eXtreme Gradient Boosting (XGBoost), have a common weakness that all the sample points for every feature are scanned when obtaining the best segmentation point; this is very time-consuming and computationally expensive to meet current needs. In order to reduce the cost of the experiment, we use LightGBM as the classifier (Friedman, 2001; Ke et al., 2017). LightGBM includes two main algorithms: Gradient-Based One-Side Sampling (GOSS) and Exclusive Feature Bundling (EFB).

In the GOSS algorithm, the training instances are firstly ranked according to the absolute values of their gradients in descending order. Then, the top-a ×100% instances with the larger gradients are kept and combined into an instance subset A. Besides, the (1−a)×100% instances with the smaller gradients are integrated in the remaining set *A^c^*, and a further subset B with the size b×|*A^C^*| is randomly sampled. Finally, the instances are split according to the estimated variance gain Vj(d)′ over the subset A⋃B. The variance gain of splitting feature *j* at point *d* is shown as follows (Ke et al., 2017)

(12) $$V^{'}{}_{j}\left(\text{d}\right)\;\frac{1}{n}\left(\frac{{\left(\sum_{x_{i}\in A_{l}}g_{i}+\frac{1-a}{b}\sum_{x_{i}\in A_{l}}g_{i}\right)}^{2}}{n_{l}{}^{j}(d)}+\frac{{\left(\sum_{x_{i}\in A_{r}}g_{i}+\frac{1-a}{b}\sum_{x_{i}\in B_{r}}g_{i}\right)}^{2}}{n_{r}{}^{j}(d)}\right)$$

where*A*_*l*_={*x*_*i*_ ∈ A:*x*_*ij*_≤d},*A*_*r*_={*x*_*i*_ ∈ A:*x*_*ij*_ > d},*B*_*l*_={*x*_*i*_ ∈ B:*x*_*ij*_≤d},*B*_*r*_={*x*_*i*_ ∈ *B*:*x*_*ij*_ > *d*}, and $\frac{1-a}{b}$ is used to normalize the sum of the gradients over *B* back to the size of *A^c^*. Each *x_i* is a vector with the dimension *s* in space *X^S^*. In every gradient boosting iteration, the negative gradients of the loss function with respect to the output of the model are defined as {*g*_1_, ^.^^.^^.^, *g*_*n*_}, where *n* is the number of vectors in space *X^S^*.

In the EFB algorithm, unnecessary computation for zero feature values is avoided by binding mutually exclusive features together in a histogram to form a feature. There are two main ideas for EFB. In algorithm 1, the function is to consider which features should be bundled together, while algorithm 2 determines how to construct the bundle as follows (Ke et al., 2017):

**ALGORITHM 1:** Greedy bundling.

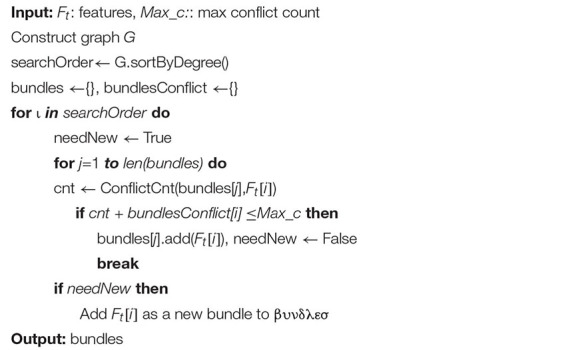		

**ALGORITHM 2:** Merge exclusive features.

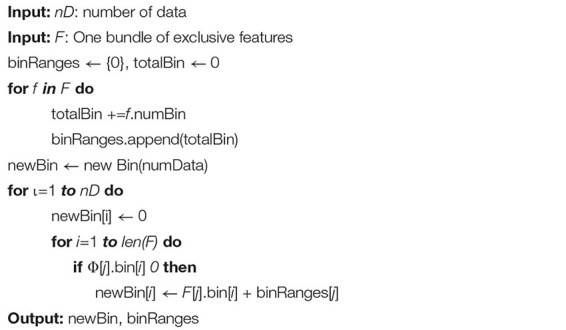		

## Results

**FIGURE 3:**
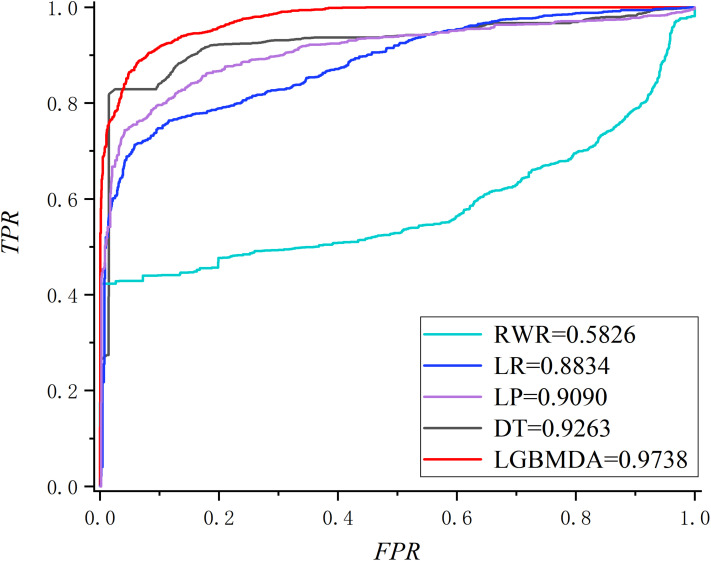
The ROC about LOOCV.

**FIGURE 4:**
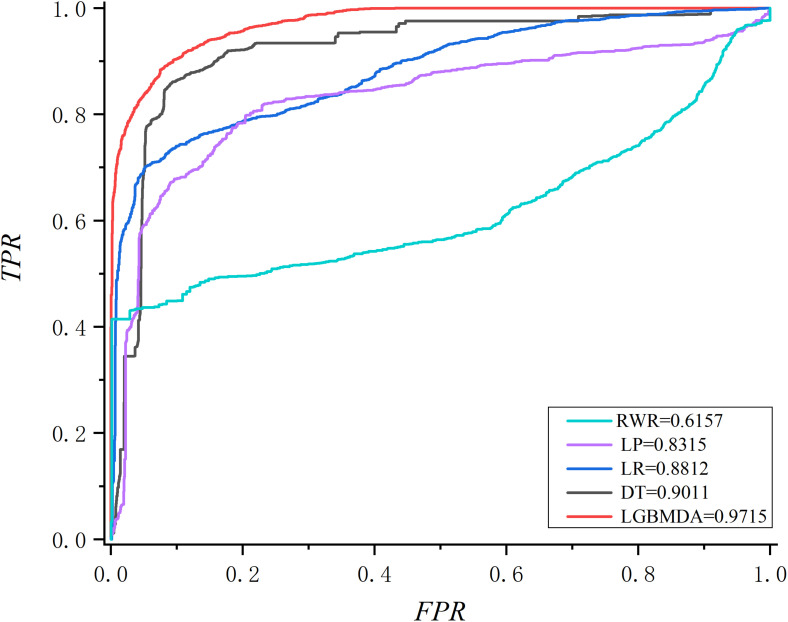
The ROC about fivefold cross validation.

**FIGURE 5:**
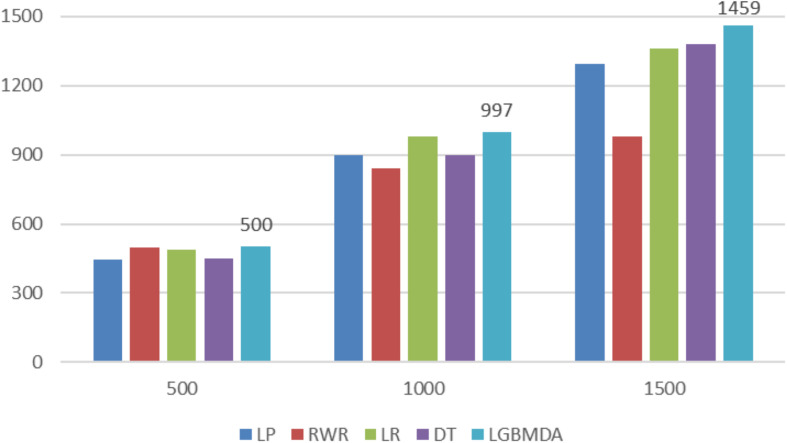
Comparison of the top *k* ranks with different methods.

**FIGURE 6:**
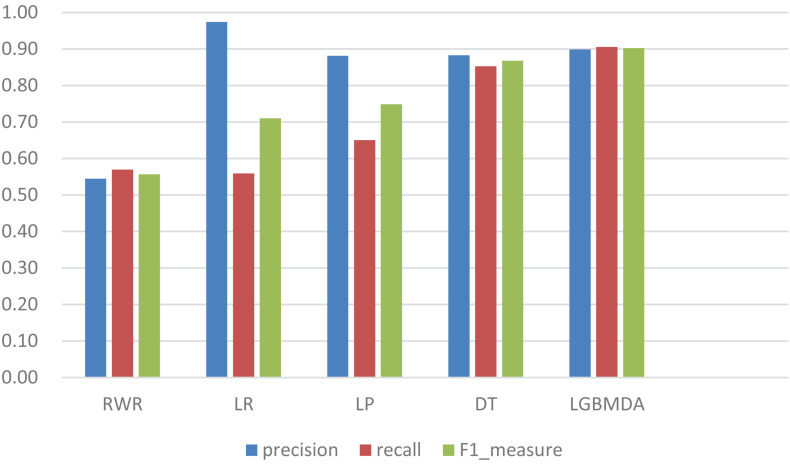
Comparison of the precision, recall, and F1_measure with different methods.

In this section, we utilize LOOCV and fivefold cross-validation to evaluate the performance of LGBMMDA. In LOOCV, each confirmed metabolite–disease pair is treated as the test set in turn, while the other confirmed pairs are regarded as training sets. Besides, the unconfirmed associations are regarded as potential candidates for true associations. We plot the ROCs curves and use the area under the ROC curve (AUC) as the evaluating indicator. Furthermore, we also use fivefold cross-validation as an evaluation tool to verify the performance of our method. In this method, the known information about metabolites and diseases is randomly divided into five equal parts. Then, each part is used as the test set in turn, while the other four parts represent the training set. This helps to avoid having the test and training data overlapping with each other and ensures unbiased comparisons. In this study, we compare our method with some state-of-the-art methods, including the label propagation algorithm (LP), which is a semi-supervised learning method based on graph (and its basic idea is to predict the label information of unlabeled nodes by using the label information of labeled nodes); random walk (RWR), which is close to Brownian motion and is the ideal mathematical state of Brownian motion; logistic regression (LR), which is a machine learning method solving binary (0 or 1) problems and estimating the possibility of something; and decision tree (DT), which is the process of classifying data through a series of rules. The results show that LGBMMDA achieved AUC values of 0.9738 and 0.9715 in LOOCV and fivefold cross-validation, respectively (see Figures 3, 4). In addition, we analyze the scores of known associations about LOOCV and count the number of known associations correctly identified by each algorithm (see Figure 5). It can be seen from Figure 6 that our proposed method is superior to other methods in terms of precision, recall, and F1-measure (0.898596, 0.90566, and 0.9021, respectively). Although the precision of LR is higher than our method, the recall of LR is significantly lower. Our method is steadier than LR.

## Parameter Analysis

**FIGURE 7:**
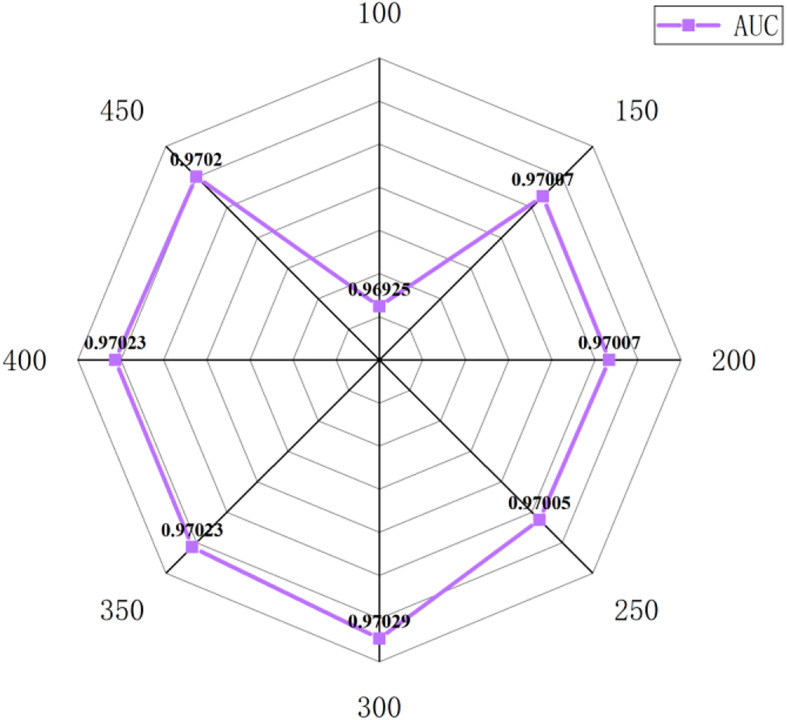
The AUC value of different *n_estimators*.

**FIGURE 8:**
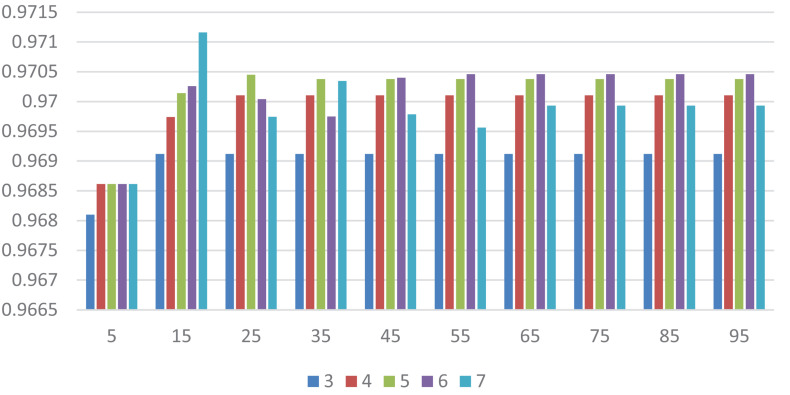
The AUC value of different *max_depth* and *num_leaves*. Different color represents different values of *max_depth.* The *X* axis represents the different values of *num_leaves*, and the *Y* axis represents relevant AUCs.

**FIGURE 9:**
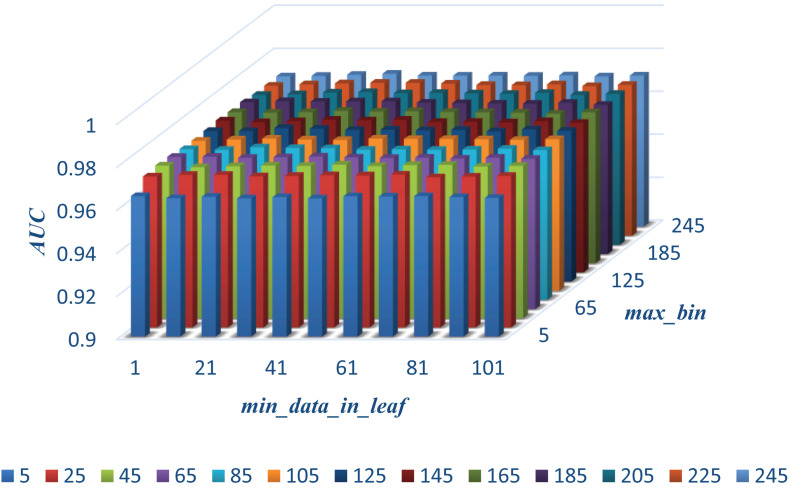
The AUC values of different max_bin and min_data_in_leaf.

In this section, we select some significant parameters to be adjusted in LightGBM. Firstly, we set the parameter *n_estimators*, which is related to the number of residual trees, from 100 to 500, while other important parameters are set to default. Figure 1 shows that we get better results when *n_estimators* is set to 300 (see Figure 7). In order to improve the accuracy, the values of the parameter *max_depth*, which limits the maximum depth of the tree model, is set from 3 to 8, and *num_leaves*, which controls the number of leaf nodes, is set from 5 to 100. As a result, *max_depth* = 7 and *num_leaves* = 15 achieve better performance (see Figure 8). Finally, the range of *max_bin*, which has an effect on overfitting, is set from 5 to 256, and *min_data_in_leaf*, which is the minimum number of samples contained on a leaf node, is set from 1 to 100. The results show that *max_bin* = 45 and *min_data_in_leaf* = 51 are better than other values (see Figure 9).

## Case Study

**FIGURE 10:**
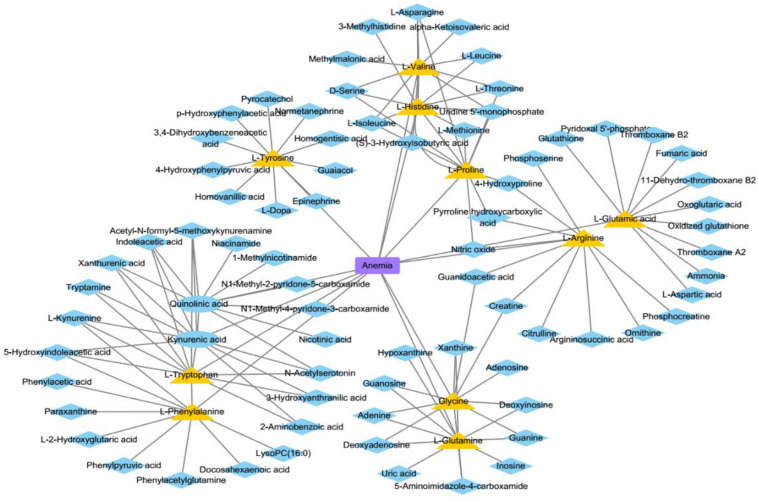
The associations between anemia and some metabolites. The blue ellipses represent the known metabolites about anemia in this study. The yellow triangles represent the top 10 predicted metabolites relevant to anemia. The blue diamonds represent the top 10 neighbors about predicted or known metabolites.

In this section, we analyze three kinds of diseases, anemia, uremia, and asthma, in case studies to discover their pathogenic mechanisms from the perspective of metabolites. There are 10, 9, and 7 metabolites of these diseases that could be verified out of the top 10 predicted metabolites, respectively. Figure 10 shows anemia and its relevant metabolites.

Anemia is caused by the inability of the body to produce enough hemoglobin, which is a protein that carries oxygen to blood cells and tissues. This disease has common symptoms, such as fatigue and dizziness. We conduct our method on a case study of anemia (see Table 1) to select the top 10 most likely associated metabolites, and all of them are associated with anemia according to literature in NCBI. For instance, L-histidine (Peterson et al., 1998) acts as a semi-essential amino acid, which is medically used in the treatment of anemia (Wang et al., 2020).

**TABLE 1:** Candidate metabolites of anemia.

**Anemia**		
**Rank**	**Metabolite name**	**Evidences**
1	L-Histidine	PMID: 32498848
2	L-Proline	PMID: 26821380
3	Glycine	PMID: 30853991
4	L-Arginine	PMID: 31355573
5	L-Valine	PMID: 30860750
6	L-Tryptophan	PMID: 32153576
7	L-Glutamine	PMID: 32350885
8	L-Tyrosine	PMID: 32764239
9	L-Glutamic acid	PMID: 30628549
10	L-Phenylalanine	PMID: 26956768

Asthma is a common and frequent disease, which has the main symptoms of paroxysmal wheezing, chest tightness, and cough. The field of metabolomics has been used to explore the metabolic signatures of asthma, both for biomarker identification and pathophysiologic mechanisms research. We perform our method on a case study of asthma, and 7 of the top 10 predicted metabolites that are interrelated with asthma are verified to be correlative (see Table 2). For example, L-proline (Nadler et al., 1988) is one of metabolic characteristics of asthma, which is supported by experimental asthma models and clinical studies in children and adults (Pite et al., 2018). Another example is L-tryptophan (Hartzema et al., 1991), which has long been suggested to be relevant to the pathophysiology of asthma (Hu et al., 2020).

**TABLE 2:** Candidate metabolites of asthma.

**Asthma**		
**Rank**	**Metabolite name**	**Evidences**
1	L-Histidine	PMID: 31206804
2	L-Proline	PMID: 29059088
3	L-Tryptophan	PMID: 31951781
4	L-Glutamic acid	–
5	3-Hydroxybutyric acid	PMID: 32213896
6	Succinic acid	PMID: 14846625
7	L-Methionine	PMID: 32778730
8	1-Methylhistidine	PMID: 24783928
9	L-Threonine	–
10	PC(18:1(11Z)/22:1(13Z))	–

Uremia is a serious kidney disease that is caused by a disorder in the internal biochemical process after renal function loss. We conduct our calculation method on a case study of uremia. As illustrated in Table 3, 9 of the top 10 predicted metabolites that are interrelated with uremia are verified to be correlative. For example, L-histidine is found to be significantly enhanced in the brain in uremia patients (Schmid et al., 1996). The L-proline in body fluids is a biological parameter for patients with renal insufficiency and chronic uremia (Hanwen, Sun et al., 2009).

**TABLE 3:** Candidate metabolites of uremia.

**Uremia**		
**Rank**	**Metabolite name**	**Evidences**
1	L-Histidine	PMID: 8676800
2	L-Proline	PMID: 20355181
3	3-Hydroxybutyric acid	
4	Biotin	PMID: 6322032
5	Xanthine	PMID: 19379356
6	L-Tryptophan	PMID: 935125
7	Inosine	PMID: 9607216
8	Succinic acid	PMID: 13837895
9	L-Glutamic acid	PMID: 6508956
10	gamma-Aminobutyric acid	PMID: 16797388

## Discussion

Uncovering complex disease-related metabolites is a vital research topic in metabolomics. To this end, we proposed a computational model called LGBMMDA under the framework of LightGBM. The experimental results by cross-validation have proven that our method outperforms previously used methods. Furthermore, three case studies indicate that the metabolite–disease correlations predicted in our method can be effectively demonstrated by relevant experiments. The LGBMMDA method is expected to be a useful biomedical research tool for predicting potential metabolite–disease associations.

There are three factors that contribute to the ideal predictive performance of LGBMMDA. Our method makes the following contributions for uncovering metabolite–disease associations: Firstly, the data of the metabolite–pathway associations are selected as metabolite functional similarities, which is a novel way to calculate similarities between metabolites. Secondly, three features are extracted by different angles, which keeps the diversity of features and contributes to a reliable performance. Thirdly, our method utilizes the reliable classifier of LightGBM, which ensures an effectively predictive accuracy.

However, there are several limitations in our prediction model. On the one hand, many parameters of GBM need to be adjusted. In this work, parameter adjustment is only carried out by some experiments. In future work, some algorithms might be used to adjust those parameters. On the other hand, more useful methods for calculating relevant similarities could be beneficial to enhancing the performance of our model. In the future, more biologically relevant information is expected to be available, which can be used to refine the similarities.

## Data Availability Statement

Publicly available datasets were analyzed in this study. This data about metabolites can be found here: https://hmdb.ca/.

## Author Contributions

CZ carried out the method IBNPLNSMDA to predict the potential associations of metabolites and diseases, participated in its design, and drafted the manuscript. XL and LL helped to draft the manuscript. All authors read and approved the final manuscript.

## Conflict of Interest

The authors declare that the research was conducted in the absence of any commercial or financial relationships that could be construed as a potential conflict of interest.

## Abbreviations

AUC: area under the curve
GIP: gaussian interaction profile
LOOCV: leave-one-out cross-validation
ROC: receiver operating characteristic.
